# Parental neural responsivity to infants’ visual attention: How mature brains influence immature brains during social interaction

**DOI:** 10.1371/journal.pbio.2006328

**Published:** 2018-12-13

**Authors:** Sam V. Wass, Valdas Noreika, Stanimira Georgieva, Kaili Clackson, Laura Brightman, Rebecca Nutbrown, Lorena Santamaria Covarrubias, Vicky Leong

**Affiliations:** 1 University of East London, London, United Kingdom; 2 Cambridge University, Cambridge, United Kingdom; 3 Nanyang Technological University, Singapore; Stanford University, UNITED STATES

## Abstract

Almost all attention and learning—in particular, most early learning—take place in social settings. But little is known of how our brains support dynamic social interactions. We recorded dual electroencephalography (EEG) from 12-month-old infants and parents during solo play and joint play. During solo play, fluctuations in infants’ theta power significantly forward-predicted their subsequent attentional behaviours. However, this forward-predictiveness was lower during joint play than solo play, suggesting that infants’ endogenous neural control over attention is greater during solo play. Overall, however, infants were more attentive to the objects during joint play. To understand why, we examined how adult brain activity related to infant attention. We found that parents’ theta power closely tracked and responded to changes in their infants’ attention. Further, instances in which parents showed greater neural responsivity were associated with longer sustained attention by infants. Our results offer new insights into how one partner influences another during social interaction.

## Author summary

We are a social species. Most infants and young children spend the majority of their early waking hours in the company of others. However, almost everything that we know about how the brain subserves early attention and learning comes from studies that examined brain function in one individual at a time just because it is easier to do experiments that way. Here, we examine the neural correlates of how attention is shared between two people engaged in social interaction. We recorded brain activity from infants and parents using scalp electroencephalogram during parallel solo play with toys and during joint play. We examined the associations between attention and brain activity in each member of the dyad independently (infant attention–infant brain, parent attention–parent brain), and we also examined cross-dyad associations (infant attention–parent brain). Our findings suggested that infants’ attention is more endogenously controlled during solo play than joint play. They also suggested that parents are neurally responsive to their infants during social play, and that, when the parent is more neurally responsive, the infant is more attentive.

## Introduction

Attention and learning are supported by endogenous oscillatory activity in the brain [1–4]. The nature of these oscillations and their relationship to behaviour develop and change from infancy into adulthood [5–9]. In infants, convergent research has suggested that theta band oscillations, which are particularly marked during early development [10], are associated with attentional and encoding processes. Theta band activity increases in infants during periods of anticipatory and sustained attention [11]; in 11-month-old infants, differences in theta band oscillations during object exploration predict subsequent object recognition during preferential looking [12]. Theta activity also increases in infants in social compared to nonsocial settings [13] and is particularly marked in naturalistic settings [13].

Although considerable previous research has investigated how brain oscillations relate to an individual’s behaviour, only a smaller body of research has investigated the neural mechanisms through which interpersonal and social factors influence behaviour [14–16]. This is despite the fact that our brains have evolved for social living [17], and most of our lives—particularly early life—are spent in social settings [18]. Understanding how social influences on attention and learning are substantiated across the brains of people engaging in social interaction, particularly during the crucial early stages of attention and learning, is an important goal for research [19, 20].

Previous work has shown that social factors influence infant attention and behaviour over short time-frames (seconds/minutes) and long timeframes (months/years). Over long timeframes, the children of parents who engage in more joint engagement during play show superior cognitive outcomes [21–23]. Over short timeframes, when an infant and social partner jointly attend to the same object during naturalistic play, infant attention is increased [24]. Recent research has contrasted two explanations for this finding: first, that social context may cause infants to be more attentive because they are more in control of their own attention behaviours. Second, that social context may offer increased opportunities for parents to scaffold their child’s attention using external attention cues—so infants are more attentive even though they are less in control of their own attention behaviours [25]. Time-series analyses conducted to evaluate these two hypotheses provided evidence more consistent with the latter hypothesis: first, infants’ rate of change of attentiveness was faster during joint play than solo play, suggesting that internal attention factors, such as attentional inertia, may influence looking behaviour less during joint play [26]. Second, adults’ attention forward-predicted infants’ subsequent attention more than vice versa [25]. These behavioural results suggest that infants’ increased attentiveness during social relative to solo play may be attributable to the presence of attention scaffolding from parents using exogenous attention cues [27]. However, to our knowledge, no previous work has examined this question from the neural perspective.

Previous research has shown that ostensive social cues such as eye gaze and vocalisations can lead to increases in interpersonal neural synchrony between infants and adults [28]. Bidirectional Granger-causal influences between the brains of infants and adults engaged in social interaction were observed in the theta and alpha frequency bands, which were stronger during direct relative to indirect gaze [28; see also 29; 30]. Infants vocalised more frequently during direct gaze, and individual infants who vocalised longer elicited stronger synchronisation from the adult [28]. These findings raise the possibility that conversely, interpersonal influences between the brains of individuals engaged in social interaction may also actively drive their partners’ attentional processes and behaviour. However, in this previous research, the direct link to attention and behaviour was not examined.

**Fig 1:**
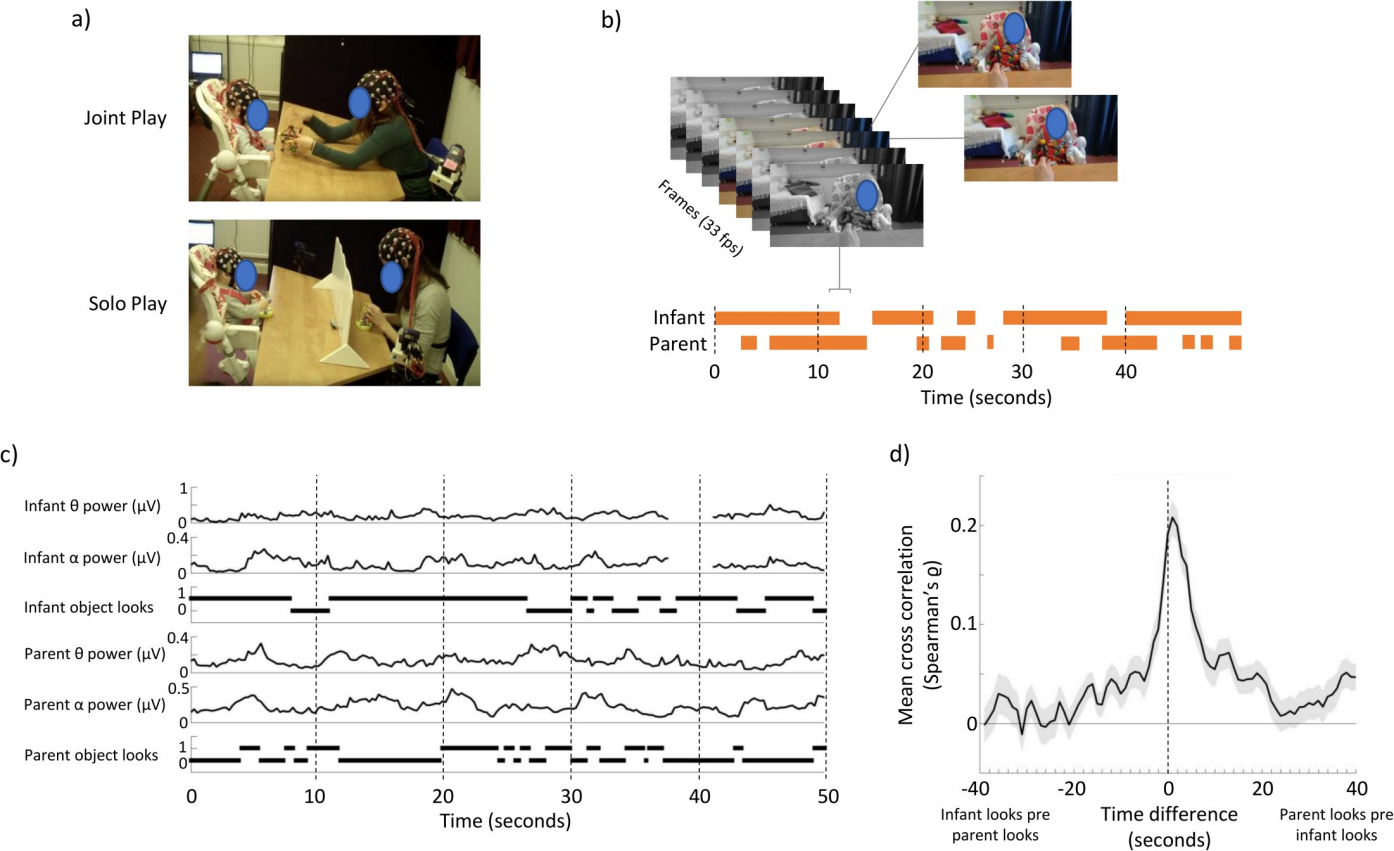
Experimental overview. (a) Demonstration of experimental set-up; (b) illustration of visual coding that was applied to the data; (c) illustration of raw data. EEG data were decomposed using a Fourier decomposition, and power within continuous bins was calculated, epoched to 4 Hz; (d) cross-correlation showing the relationship between infant object looks and parent object looks [see 25]. The underlying data for this figure can be found in S1 Text. EEG, electroencephalography.

Here, we examined the neural and behavioural dynamics of infants’ and adults’ attention in two contexts (see Fig 1). During joint play, each dyad was presented consecutively with toy objects and asked to play together. During solo play, a 40-cm-high divider was placed between the infant and the parent, and two identical toys were presented concurrently to child and parent, who played separately (see Fig 1). Looking behaviour was videoed and coded post hoc, frame by frame, at a rate of 30 Hz. Time-lagged cross-correlations were used to assess how changes in one time series preceded or followed changes in another [31; cf. 32, 33]—an approach similar, but not identical, to Granger causality [34]. Our analyses examined whether changes in one time series ‘forward-predicted’ changes in the other. The age of the infants was selected to be 12 months because this is considered the age at which the capacity for endogenous control of attention first starts to develop rapidly [35, 36]. As is typical [e.g., 24], visual attention was coded as the presence or absence of looking behaviour towards the play object—albeit that previous research has shown the limitations of looking behaviour alone as an index of attention [37, 38, 39].

Based on previous research [10, 13], we expected that fluctuations in infant theta activity would associate with and forward-predict fluctuations in infant attentiveness. Based on our previous research [25], we predicted that the forward-predictive relationship between infants’ own endogenous brain activity and infants’ attentiveness would be higher during solo play than joint play because of the increased prevalence of exogenous parental attention scaffolding (and capture) during joint play. Further, since previous research indicates that parental responsiveness is an influential factor for early developing cognition [40, 41], we also examined the short-term associations between infants’ attention and neural activity in the parent. We predicted, in the absence of prior investigations in this area, that a higher association between infant attention and neural activity in the parent would predict greater attentiveness from the infant.

## Results

Analysis 1 examines the within-individual relationship between electroencephalography (EEG) power and visual attention separately for joint play and solo play. Analysis 2 examines the cross-dyad relationship between parent EEG power and infant visual attention separately for joint play and solo play. Analysis 3 examines changes in EEG power relative to individual look onsets. This was also calculated both within individual and across dyad.

### Analysis 1: Cross-correlation, within participant

**Fig 2:**
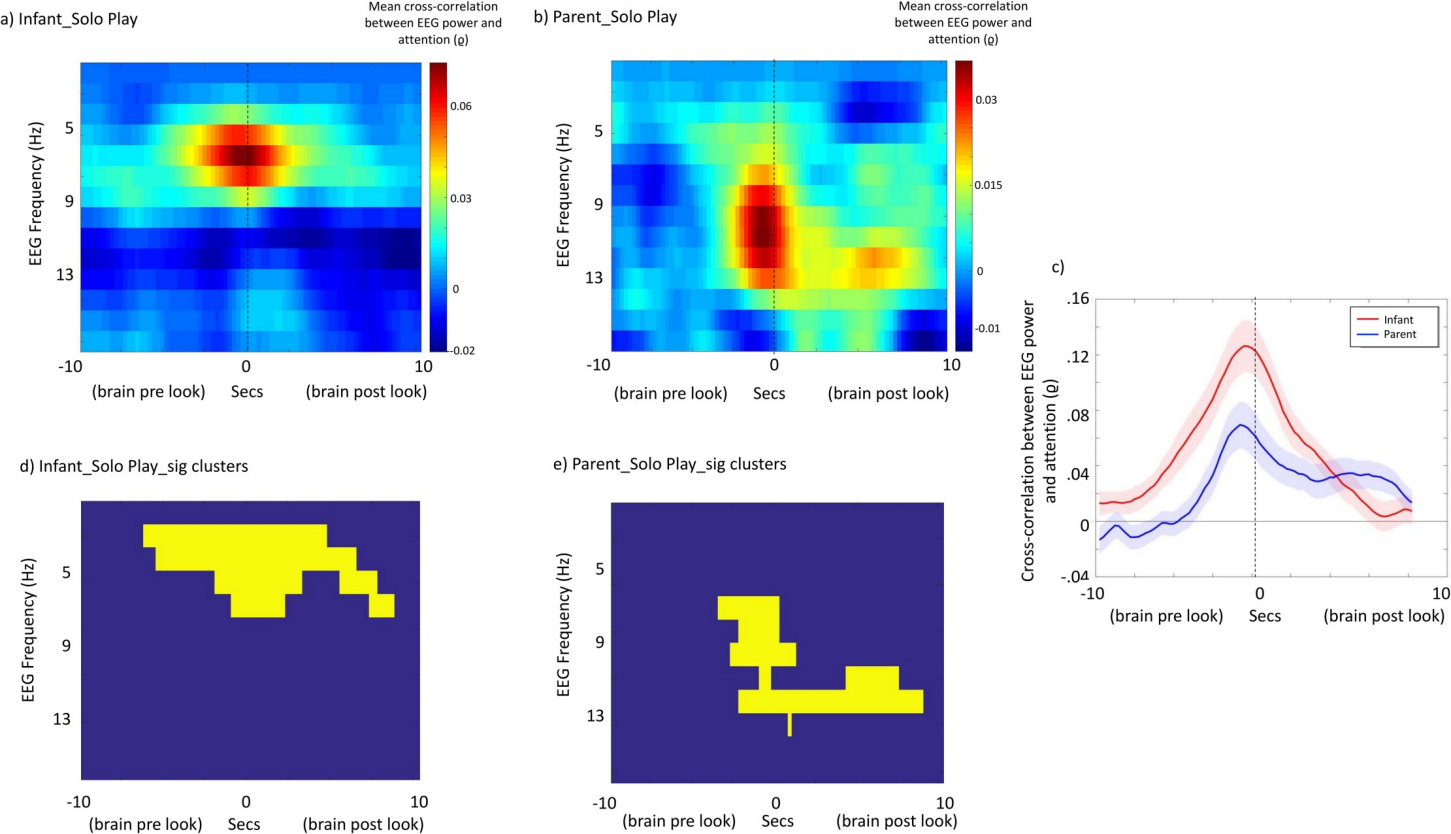
Brain–behaviour associations: Solo play. (a and b) Mean time-lagged cross-correlations between EEG power and visual attention for (a) infant solo play and (b) parent solo play. Time lag between EEG power and visual attention is shown on the x-axis, and the EEG frequency on the y-axis. (c) Cross-correlation plots just for those frequency bands identified from the cluster-based permutation test as showing the most marked differences from chance (infant: 3 Hz–7 Hz; adult: 6 Hz–12 Hz). x-axis shows time; y-axis, cross-correlation between EEG power and attention. Shaded areas show the standard error of the means. (d and e) Results of the cluster-based permutation statistic. Yellow squares indicate time × frequency points of significant cross-correlations. The underlying data for this figure can be found in S1 Text and S1 Data.

Fig 2 shows time-lagged cross-correlations between EEG power and visual attention for solo play. Fig 2A and 2B show correlations across the frequency spectrum, with time-lag on the x-axis and EEG frequency on the y-axis. Fig 2C and 2D show results of the cluster-based permutation test. These suggested that the results for both infant solo play (*p* = 0.002) and adult solo play (*p* = 0.002) differed significantly from chance. For infants, the effect was most pronounced in the 3 Hz–7 Hz range (Fig 2D); for adults, in the 6 Hz–12 Hz range (Fig 2E). In addition, to further confirm the results, a separate bootstrapping analysis was conducted as described in the S1 Text (section 2.vi), which yielded identical results.

In order to examine at which time window the peak cross-correlation was observed between EEG power and visual attention, we excerpted the cross-correlation values just for those frequency bands identified from the cluster-based permutation test (infants: 3 Hz–7 Hz; adults: 6 Hz–12 Hz; see Fig 2C). For infants, the peak cross-correlation was observed at *t* = –750 ms (i.e., between EEG power at time *t* and attention 750 ms after time *t*). For adults, the peak cross-correlation was observed at *t* = –1,000 ms. (Of note, these numbers do not indicate the time lag of the EEG data relative to the onset of a look but rather the time lag of the largest cross-correlation between EEG power and attention when treated as two continuous variables.)

**Fig 3:**
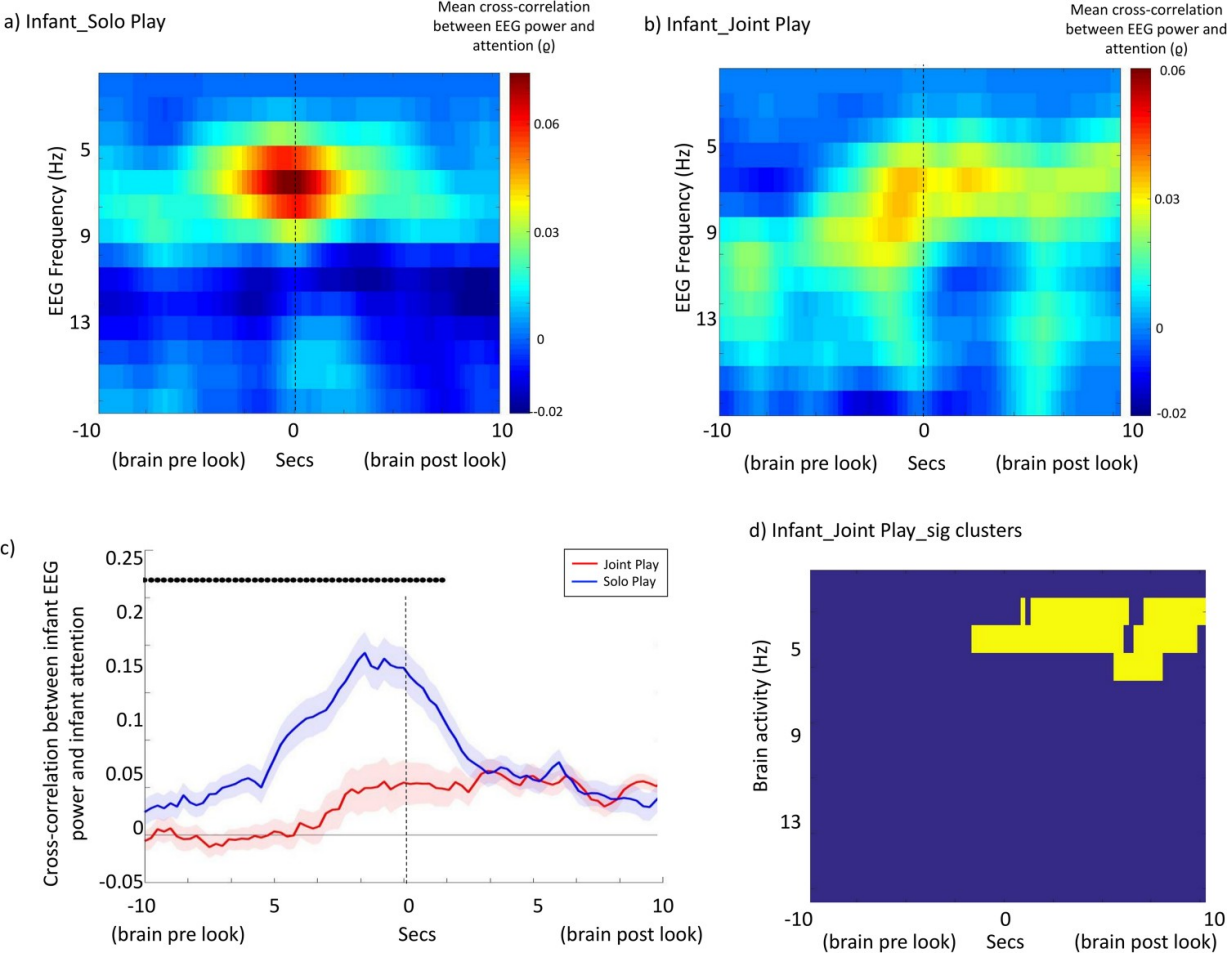
Brain–behaviour associations: Infant solo play and joint play. (a and b) Mean time-lagged cross-correlations between EEG power and visual attention for (a) infant solo play and (b) infant joint play. (Fig 3A is identical to Fig 2A but included to allow for comparison with Fig 3B). (c) Line plot showing cross-correlation between EEG power and visual attention for just the frequency ranges identified from the cluster-based permutation test as showing marked effects in both conditions (3 Hz–6 Hz). Red shows the joint play condition, and blue the solo play condition. Shaded areas show interparticipant variance (standard errors). Dots above the plots indicate the results of the significance calculations to assess whether the correlations observed differed significantly between the two conditions. (d) Results of the cluster-based permutation statistic for infant joint play. Yellow squares indicate time × frequency points of significant cross-correlations. The underlying data for this figure can be found in S1 Text and S1 Data.

Fig 3 compares the mean time-lagged cross-correlations for infant solo play and infant joint Play. All data, including unpaired data, have been included (see Participants). Fig 3A and 3B show cross-correlation plots across the frequency spectrum. (Fig 3A is identical to 2a and included to allow comparison with Fig 3B) Fig 3D shows the cluster-based permutation test for the infant joint play condition. This suggested that the infant joint play condition differed significantly from chance (*p* = 0.008).

To directly compare the peak cross-correlation values obtained for infant solo play and infant joint play, we excerpted the cross-correlation values just for those frequencies that the cluster-based permutation test indicated as showing marked differences in both conditions (3 Hz–6 Hz; see Fig 2C). For solo play, the peak cross-correlation was at *t* = –1,500 ms (EEG power at time *t* to attention 1,500 ms after time *t*); for joint play, the peak cross-correlation was at *t* = +3,000 ms.

In addition, separate unpaired *t* tests were conducted at each time window to compare the results across conditions and adjusted for multiple comparisons using the Benjamini–Hochberg false discovery rate procedure [42]. Time windows showing significant differences are indicated using black dots above the plot in Fig 3C. Results indicate that larger cross-correlations were observed during solo play relative to joint play for all time lags between *t* = –10,000 ms and *t* = +1,250 ms.

**Fig 4:**
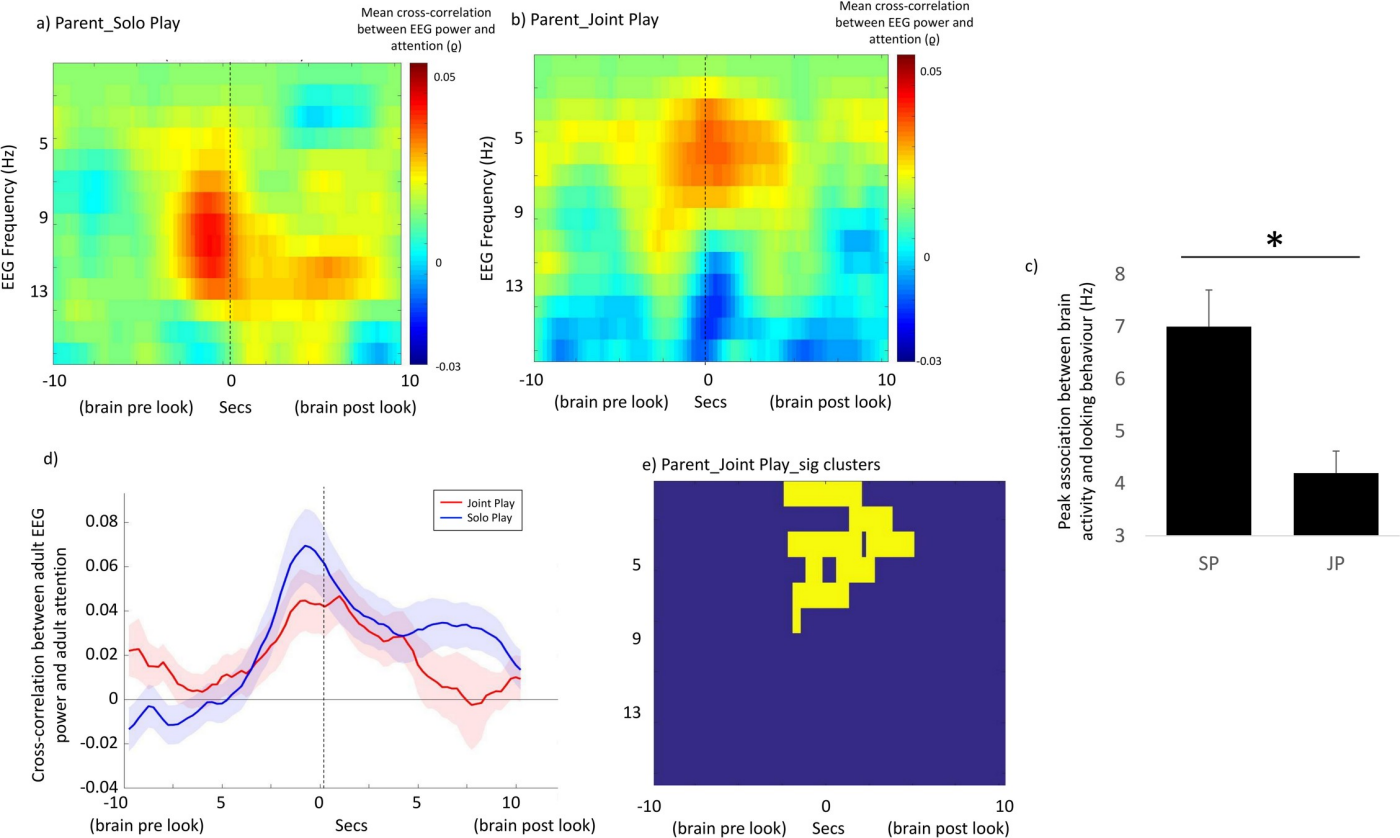
Brain–behaviour associations: Adult solo play and joint Play. (a and b) Mean time-lagged cross-correlations examining the relationship between EEG power and attention for parent solo play and parent joint play. (Fig 4A is identical to Fig 2B but scaled to be equivalent to Fig 4B to allow for comparison.) (c) Bar chart comparing the frequency of the peak association between EEG power and looking behaviour for parents in the solo play and joint play conditions. * indicates the results of the significance calculations, conducted as described in the main text. (d) Line plot showing cross-correlation between EEG power and visual attention for just the frequency ranges identified from the cluster-based permutation test as showing marked effects in both conditions (parent solo play: 6 Hz–12 Hz; parent joint play: 2 Hz–8 Hz). Red shows the joint play condition, and blue the solo play condition. Shaded areas show interparticipant variance (standard errors). (e) Results of the cluster-based permutation statistic for parent joint play. Yellow squares indicate time × frequency points of significant cross-correlations. The underlying data for this figure can be found in S1 Text and S1 Data.

Fig 4A and 4B show the mean time-lagged cross-correlations for parent solo play and parent joint play. Fig 4E shows the cluster-based permutation test for parent joint play, which indicated significant differences from chance (*p* = 0.001). For parent solo play, the most marked associations between EEG power and attention were at 6 Hz–12 Hz (Fig 2B); for parent joint play, the most marked associations were at 2 Hz–8 Hz (Fig 4E). To assess the significance of this difference, we measured the frequency of peak association between EEG power and attention for parents during solo play and joint play across all frequency bands under consideration (2 Hz–12 Hz) during the ±1,000 ms time window. Results obtained from the two conditions were compared using a paired *t* test; a significant difference between the two conditions was observed (*t*(44) = 3.42, *p* = 0.001). This suggests that the peak association between brain activity and attention in the parent was observed at lower frequencies during joint play than during solo play.

### Analysis 2: Cross-correlation across parent and infant

**Fig 5:**
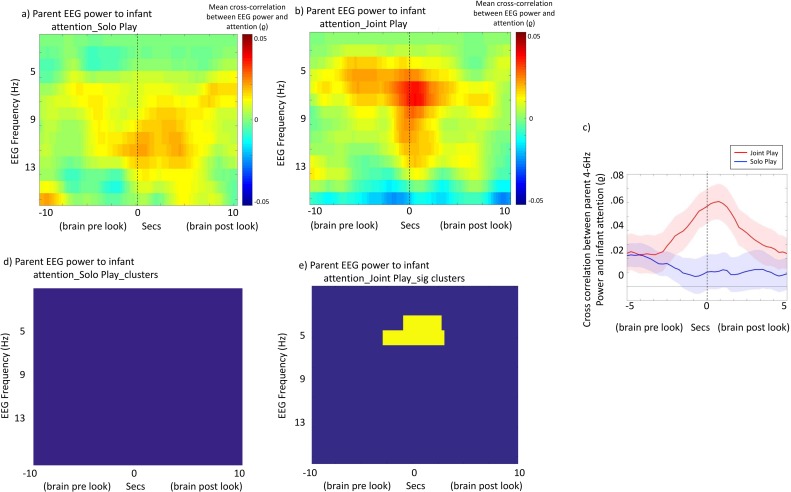
Brain–behaviour associations: Adult brain and infant behaviour. (a and b) Mean time-lagged cross-correlations between parent EEG power and infant attention for (a) solo play and (b) joint play. Time lag between brain activity and visual attention is shown on the x-axis, and the EEG frequency on the y-axis. (c) Line plot showing cross-correlation between EEG power and visual attention for just the frequency ranges identified from the cluster-based permutation test as showing marked differences in the joint play condition (4 Hz–6 Hz). Red shows the joint play condition, and blue the solo play condition. Shaded areas show interparticipant variance (standard errors). (d and e) Results of the cluster-based permutation statistic. Yellow squares indicate time × frequency points of significant cross-correlations. The underlying data for this figure can be found in S1 Text and S1 Data.

Fig 5A and 5B show the mean time-lagged cross-correlations, and Fig 5D and 5E show the cluster-based permutation tests, for the relationship between parents’ EEG power and infants’ attention. For parent EEG and infant attention in the joint play condition, a significant relationship was identified (*p* = 0.041). The most marked associations were identified in the 4 Hz–6 Hz range (Fig 5E). An identical analysis examining the relationship between parent EEG and infant attention in the (concurrent but separate) solo play condition identified no significant relationship. In addition, a further bootstrapping analysis was performed (see S1 Text), which confirmed that the observed cross-correlation values significantly exceed chance for joint play but not solo play.

For the within-participant analysis of solo play, the peak cross-correlation values observed were consistently negative (‘brain pre-look’) (Figs 2C and 3C). In order to directly compare the peak cross-correlation values obtained between the solo play and joint play conditions, we excerpted the cross-correlation values just for those frequency bands identified from the cluster-based permutation test as showing marked differences during joint play (4 Hz–6 Hz) (see Fig 5C). For joint play, the peak cross-correlation value occurred at a *t* = +750 ms (i.e., between infant attention at time *t* and adult EEG 750 ms after time *t*, ‘adult brain post-infant look’).

### Analysis 3: Calculation of power changes around looks

In addition, we conducted a further analysis using separate procedures from those used in Analyses 1 and 2. Whereas Analyses 1 and 2 examine the cross-correlation between EEG power and attention when treated as two continuous variables, Analysis 3 examines changes in EEG power relative to the onsets of individual looks.

We examined all looks to the play objects that occurred during the session. For each look, we excerpted the power in the theta band for three time windows immediately prior to the onset of each look (3,000–2,000, 2,000–1,000, and 1,000–0 ms pre-look onset) and three windows immediately after the onset of each look (0–1,000, 1,000–2,000, and 2,000–3,000 ms post look onset). Theta power was defined according to the frequency bands identified from the cluster-based permutation tests as showing the most marked differences from chance. These were infant solo play (Fig 2D)—3 Hz–7 Hz; infant joint play (Fig 3D)—4 Hz–7 Hz); adult to infant (Fig 5E)—4 Hz–6 Hz.

**Fig 6:**
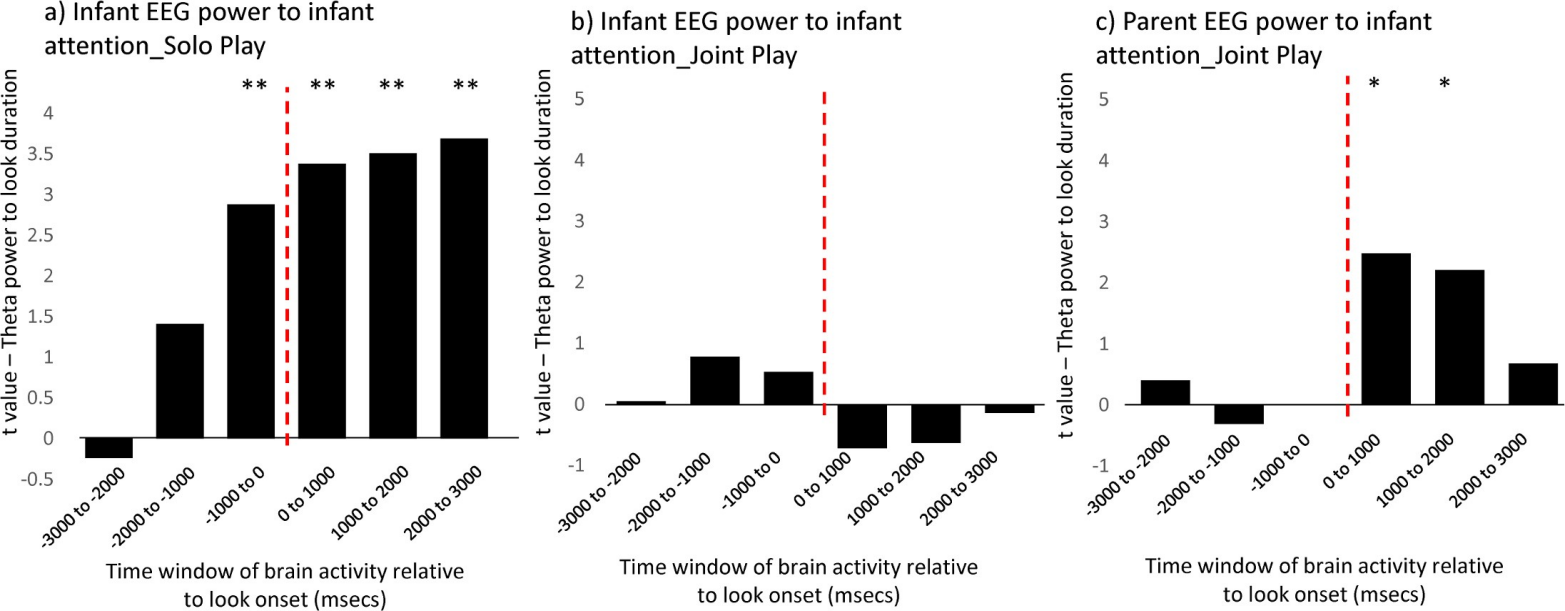
Analysis 3 results. Results of linear mixed effects models conducted to examine whether individual looks accompanied by higher theta power are longer lasting. For each look, the theta power for three time windows prior to look onset (3,000–2,000, 2,000–1,000, and 1,000–0 ms pre-look) and for three time windows post look onset (0–1,000, 1,000–2,000, and 2,000–3,000 ms post-look) was excerpted. We then calculated separate linear mixed effects models for each of the six windows to examine the relationship between EEG power within that time window and look duration. y-axis shows the *t* value. * indicates the p values (*p < 0.05, **p < 0.01). Full results are shown in S1 Table. The underlying data for this figure can be found in S1 Text and S1 Data.

We then calculated separate linear mixed effects models for each of the six windows to examine the relationship between EEG power within that time window and look duration. Full results are shown in S1 Table, and key results are shown in Fig 6. In the solo play condition (Fig 6a), a relationship was observed between infants’ theta power and look duration, consistent with the results of Analysis 1 (Fig 2A). Theta power in the time window –1,000 to 0 ms prior to look onset significantly predicted the subsequent duration of that look, consistent with the forward-predictive relationship noted in Fig 2C. The strength of this relationship increased for time windows after the onset of the look. Conversely, for joint play (Fig 6B), there was no significant relationship between infants’ theta power and look duration. Again, this finding is consistent with the results of Analysis 1 (Fig 3C).

During joint play, parental theta power associated significantly with infant attention in the time windows after the onset of the look (0–1,000 ms and 1,000–2,000 ms; Fig 6C). However, there is no relationship in the time windows prior to look onset. This result is also consistent with the results of Analysis 2 (Fig 5C).

## Discussion

It is well established that attention and learning are supported by the endogenous oscillatory neural activity of the person attending. However, relatively little is known about how interpersonal and social influences on attention are substantiated in the brain [16, 43]. To investigate this, we examined how the oscillatory dynamics of attention are shared between infant–parent dyads and how these dynamics differ between noninteractive and interactive social play.

We found that when infants were engaged in solo play, continuous fluctuations in theta power forward-predicted visual attention in infants (Fig 2). Consistent with this, a separate analysis identified a positive association between theta power in the 1,000 ms prior to look onset and the subsequent duration of that look (Fig 6). For adults, a similar functional relationship was observed but at a higher frequency (6 Hz–12 Hz) in the alpha band, consistent with considerable previous research into the role of prestimulus alpha activity in anticipatory visual attention [44, 45]. Our infant findings are also consistent with previous research suggesting that theta oscillations increase during anticipatory and sustained attention and encoding [10; 12, 13], but they are novel insofar as we demonstrated these effects during spontaneous attention in seminaturalistic settings.

During interactive social play, however, we found that this forward-predictive relationship between infants’ endogenous theta activity and visual attention was still present but much reduced. Again, this result was observed consistently across two separate analyses (Fig 3 and Fig 6). Particularly of interest was Fig 3C, which suggested that negative-lag relationships (attention forward-predicting EEG power) were similar across the solo and joint play conditions but that positive-lag relationships (EEG power forward-predicting attention) were present only during solo play. These results are consistent with our previous research suggesting that endogenous factors, such as attentional inertia, influence infants’ attention more during solo (noninteractive) play than during joint play [25]. Taken together, our results suggest that infants’ endogenous neural control over attention is greater during solo play.

These results appear unlikely to be attributable to oculomotor artefact associated with the onsets and offsets of looks for a number of reasons. First, during data preprocessing, we removed oculomotor artefacts via independent component analysis (ICA) (see S1 Text); second, we have only reported data in this paper from two channels near the vertex—C3 and C4, which show the least contamination by muscle and motion artefacts. (See S5 and S6 Figs for comparable plots of anterior and posterior midline groups.) Third, the cross-correlation analysis across different frequencies (Fig 2A) indicated that relationships were specific to the theta band. Muscular artefacts generally produce the highest contamination in delta, beta, and gamma bands [46, 47]. Fourth, effects were present around the onsets of looks in the solo play but not the joint play condition (Fig 3A and 3B).

Our findings are also unlikely to be attributable to differences in mean look duration between the two conditions (see S1 Fig) for two reasons. First, as in Analysis 1, any artefactual effects would be random rather than directional (i.e., specifically affecting negative rather than positive lags). Second, Analysis 1 examined the relationship between attention and EEG power considered across continuous entire time series, whereas Analysis 3 examined power changes relative to the onsets of individual looks, and the results from the two analyses produced converging conclusions. Furthermore, this result is also not attributable to differences in relative power between the two conditions because the EEG power spectrum of infants did not differ across conditions (S2 Fig).

Overall, however, we found that despite the fact that infants’ endogenous attention control over their own behaviour patterns appeared to be lower, they were more attentive towards objects during joint play (S1 Fig)—a finding consistent with previous research [24]. To understand why, we examined how adult brain activity related to infant attention.

First, we found that during joint play, the frequency of adults’ peak association between EEG power and attention was down-shifted to the theta range—similar to infants’ peak frequency of association (Fig 4). Second, we found that parent EEG theta power significantly tracked infant attention. Again, this result was observed across two separate analyses. Analysis 2 (Fig 5D and 5E) suggested that infant attention associated, over a time-frame of ±2 seconds, with increased parental theta power. Analysis 3 (Fig 6C) suggested that individual infant attention episodes accompanied by greater parental EEG power were longer lasting.

Importantly, we found that the direction of the peak association differed between solo and interactive play. During solo play, the peak cross-correlation between infant theta power and infant attention was observed at negative lag (‘brain pre-look’) (Figs 2C and 3C), and theta power 1,000 ms prior to look onset predicted look durations (Fig 6C). During joint play, the peak cross-correlation between adult theta power and infant attention was observed at positive lag (‘brain post-look’) (Fig 5C), and Analysis 3 identified backwards-predictive but not forward-predictive relationships between adult theta power and infant look duration (Fig 6C). These findings appear to suggest that, during joint play, parents’ theta power tracks and responds to changes in infants’ attention.

One possible account of our findings we considered is that infant attention may (Granger-) cause adult attention, which in turn causes increased theta activity in adults. This explanation appears unlikely, however, because in S1 Text, we report a control analysis in which instances in which an attention shift from the infant was immediately followed by an attention shift from the parent were excluded. The results obtained from this subset of the data were highly similar to those reported in the main text (see S8 Fig). Furthermore, as we show in Fig 1D, adults’ gaze forward-predicted infants’ attention more than vice versa, which also appears inconsistent with this explanation.

Overall, then, our results suggest that adults show neural responsivity to the behaviours of the child, and that increased parental neural responsivity associates, look by look, with increased infant attentiveness. Temporally fine-grained patterns of parental responsivity to infants have previously been shown using methods other than neuroimaging, such as microcoding of facial affect [48, 49], autonomic physiology [50], visual attention [51], and vocalisations [52; 53]. And, using neuroimaging, research with adults has provided evidence for common activation elicited when experiencing emotions such as disgust [54], touch [55], or pain [56] in oneself and when perceiving the same feelings in others. However, this is the first study, to our knowledge, to demonstrate temporal associations between infants’ attentiveness and parental neural correlates of attention and to show that moment-to-moment variability in adults’ neural activity associates with moment-to-moment variability in infants’ attentiveness.

Although demonstrated here in the context of parent–child interaction, future research should explore whether our present findings extend to cover other aspects of social interaction [57]. They should also be extended to explore individual differences—whether some social partners show greater neural responsiveness to others and how this influences behaviour [49]—and to other aspects of interpersonal neural influences than shared attention during joint play. Finally, future work should examine the mechanisms through which the children of parents who show increased responsivity over shorter timeframes develop superior endogenous attention control over long timeframes [21–23, 58, 59].

## Methods

### Ethics statement

The study was conducted according to guidelines laid down in the Declaration of Helsinki, with written informed consent obtained from a parent or guardian for each child before any assessment or data collection. All procedures involving human subjects in this study were approved by the Psychology Research Ethics Committee at the University of Cambridge (Number PRE.2016.029). No financial inducements were offered other than the reimbursement of travel expenses and the gift of a T-shirt for participating infants.

### Participants

Twenty-four and twenty-five parents contributed usable data for the joint play and solo play conditions, respectively; for infants, it was 21 and 25 for joint play and solo play, respectively. Paired parent–child data were available for 20 dyads for joint play (10 M and 10 F infants; mean [SE] infant age 345.1 [12.1] days; mother age 34.7 [0.8] years) and for 22 dyads for solo play (12 M and 10 F infants; mean [SE] infant age 339.2 [10.3] days; mother age 34.1 [1.0] years). All participating parents were female. It should be noted that the recruitment area for this study, Cambridge, United Kingdom, is a wealthy university town, and the participants were predominantly Caucasian and from well-educated backgrounds and so do not represent an accurate demographic sample [60].

### Experimental set-up

As previously reported [25], infants were seated in a high chair, which was positioned immediately in front of a table. The toys on the table were within easy reach (see Fig 1). Parents were positioned on the opposite side of the 65-cm-wide table, facing the infant. In the solo play condition only, a 40-cm-high barrier was positioned across the middle of the table (see Fig 1A). When the barrier was in place, parent and child had line of sight to one another (to reduce the possibility of infant distress), but neither could see the objects with which the other was playing.

Each infant–parent dyad took part in both the joint play and solo play conditions. Presentation order was randomised between participants, but the two conditions were presented consecutively, with a short break in between. Parents were informed that the aim of the study was to compare behaviour while they were attending to objects separately from each other and when they were attending to the same object. During the solo play condition, parents played silently with the toys alone. During the joint play condition, they played silently with the toys whilst involving their infant in the play.

A research assistant was positioned on the floor out of the infant’s sight. The research assistant placed the toys onto the table one at a time. In the joint play condition, one toy was presented at a time. In the solo play condition, two identical toys were presented concurrently to the infant and parent, one on either side of the barrier. The toys were small (<15 cm), engaging objects. Presentation order was randomised between conditions and between participants. Approximately every two minutes, or more frequently if the child threw the object to the floor, the current toy object was replaced with a new object. The mean (SE) duration for which each object was presented was 140.1 (17.9) seconds for joint play and 110.3 seconds (7.9) for solo play. Approximately 10 minutes of data was collected per condition from each dyad. The mean (SE) duration of play for each condition was 10.80 (0.46) minutes for joint play and 10.35 (0.33) minutes for solo play. When the infant became fussy during testing, data collection was stopped earlier; however, this occurred fairly rarely: the number of infants contributing sessions that lasted less than 8 minutes was 2/3 for the joint play/solo play conditions.

### Video coding and previous behavioural findings

Play sessions were videoed using two camcorders positioned next to the child and parent, respectively. Further details of video coding and synchronisation are given in S1 Text. The visual attentional patterns of parents and infants were manually coded by reviewing their respective video recordings on a frame-by-frame basis (30 frames per second, 33.3 ms temporal acuity) using video editing software (Windows Movie Maker) (see Fig 1). This coding identified the exact start and end times of periods during which the participant was looking at the toy object.

A previous report based on these data, which contained behavioural findings only, reported that infants showed longer look durations towards the object during joint play relative to solo play, together with shorter periods of inattention (see S1 Fig) [25].

### EEG data acquisition

EEG signals were obtained using a 32-channel wireless Biopac Mobita Acquisition System (Biopac Systems, Goleta, CA, USA) and 32-channel Easycap. Further details of EEG acquisition are given in S1 Text.

### EEG artefact rejection and preprocessing

Automatic artefact rejection followed by manual cleaning using ICAs was performed. Full descriptions are given in S1 Text. Because previous analyses have shown that movement and muscle artefacts can contaminate EEGs [46, 47], data from all channels other than the two channels close to the vertex, C3 and C4, were excluded, and only frequencies between 2 and 14 Hz were examined. Analyses suggested that these frequencies show the least EEG signal distortion due to sweating, movement, or muscle artefact [46]. Prior literature [e.g. 11, 61] suggests that these frequencies were also most likely to show associations with visual attention. In S5 and S6 Fig, we also include comparison plots based on alternative anterior and posterior midline electrode groupings, which are consistent with the results reported in the main text.

### EEG power analysis

For each electrode, we computed the Fourier transform of the activity averaged over artefact-free epochs, using the fast Fourier transform algorithm implemented in MATLAB (The MathWorks, Natick, MA, USA) (see S1 Text for full description). The FFT was performed on data in 2,000 ms epochs, which were segmented with an 87.5% (1,750 ms) overlap between adjacent epochs. Thus, power estimates of the EEG signal were obtained with a temporal resolution of 4 Hz and a frequency resolution of 1 Hz. S2 Fig compares EEG power for infants and parents between solo play and joint play; no significant between-condition differences were observed.

### Calculation of time-lagged cross-correlation

The attention data used for the cross-correlation analysis were resampled as continuous and time-synchronised data streams at 4 Hz (to match that of the EEG power estimate). Attention data were coded as 1 and 0 (either attentive towards the play object or not). The cross-correlation calculations were performed separately for each frequency band (in 1 Hz bands) and for each member of the dyad (infant brain–infant attention and parent brain–parent attention) (Analysis 1). Then, they were calculated across the dyad (parent brain–infant attention) (Analysis 2).

For each computation, the zero-lag correlation was first calculated across all pairs of time-locked (i.e., simultaneously occurring) epochs, comparing the EEG power profile with the attention data using a nonparametric (Spearman’s) correlation. In S4 Fig, we also show the results of the same tests repeated using an alternative test, the Mann–Whitney U test, for which results were identical.) The mean correlation value obtained was plotted as time ‘0’ (*t* = 0) in the cross-correlation. Next, time-lagged cross-correlations were computed at all lags from –10 to +10 seconds in lags of ±250 ms (corresponding to one data point at 4 Hz). For example, at lag time *t* = –250 ms, the EEG power profile was shifted one data point backwards relative to the attention data, and the mean correlation between all lagged pairs of data was calculated. Based on an average of 10.5 minutes of data per condition, sampled at 4 Hz and allowing for some attrition at artefact rejection due to the max-min thresholding criteria, the N of the cross-correlation was approximately 2,300 for the zero-lag correlation and up to 40 fewer for the most shifted correlation. In this way, we estimated how the association between two variables changed with increasing time lags. The individual cross-correlation series were then averaged across participants to obtain the group mean cross-correlation at each time interval and frequency band.

To compare the distribution of time × frequency data between any single condition and a null distribution, a cluster-based permutation test was conducted across time × frequency data using the FieldTrip function ft_freqstatistics [62]. In comparison to other approaches to solving the family-wise error rate, this approach identifies clusters of neighbouring responses in time/frequency space [63]. In particular, corresponding time × frequency points were compared between contrast condition and null distribution with a *t* test, and *t* values of adjacent spatiotemporal points with *p* < 0.05 were clustered together with a weighted cluster mass statistic that combines cluster size and intensity. The largest obtained cluster was retained. Afterwards, the whole procedure, i.e., calculation of *t* values at each spatiotemporal point followed by clustering of adjacent *t* values, was repeated 1,000 times, with recombination and randomised resampling before each repetition. This Monte Carlo method generated an estimate of the *p* value representing the statistical significance of the originally identified cluster compared to results obtained from a chance distribution.

In addition, a supplementary analysis was conducted using bootstrapping in order to further verify our results (see S1 Text).

### Calculation of power changes around looks

Analysis 3 examined whether individual looks accompanied by higher theta power are longer lasting. To calculate this, we examined all looks to the play objects that occurred during the play session. The onset times of these looks were calculated, as described above, at 30 Hz. Then, for each look, we excerpted the EEG power for three time windows immediately before and after the onset of each look (3,000–2,000, 2,000–1,000, and 1,000–0 ms pre-look onset; 0–1,000, 1,000–2,000, and 2,000–3,000 ms post-look onset).

Separately, we calculated the duration of each look towards the object. Since these were heavily positively skewed, as is universal in looking time data [64], they were log-transformed. Then, we calculated separate linear mixed effects models for each of the six windows using the *fitlme* function in MATLAB. For each model, we examined the relationship between EEG power within that time window and look duration, controlling for the random effect of participant. In this way, we examined whether, for example, theta power in the time window 1,000–0 ms prior to the onset of a look showed a significant relationship to the subsequent duration of that look.

## Supporting information

S1 Fig Attention duration data obtained for the joint play and solo play conditions. (a) Mean durations of attention episodes towards the object and inattention. Error bars show standard errors. Stars above the plots indicate that attention durations towards the object were found to be significantly longer during joint play than solo play, and episodes of inattention were significantly shorter. (b) Histogram of all attention episodes towards the object in joint play and solo play. (c) histogram of all episodes of inattention in joint play and solo play. Data underlying this figure can be found in S2 Data.
(PNG)

S2 Fig Comparison of the differences in relative power, for infants and parents, in the joint play and solo play conditions. Data underlying this figure can be found in S2 Data.
(PNG)

S3 Fig Figure showing the cross-spectrum zero-lagged cross-correlations between different individual EEG frequency bands. Data underlying this figure can be found in S2 Data. EEG, electroencephalography.
(PDF)

S4 Fig Comparison of analyses presented in the main text with results of the Mann–Whitney U test. S3A and S3B Fig—equivalent to Fig 3A and 3B. S3C and S3D Fig—equivalent to Fig 4A and 4B. S3E and S3F Fig—equivalent to Fig 5A and 5B. Data underlying this figure can be found in S2 Data.
(TIF)

S5 Fig Time-lagged cross-correlations between EEG power and visual attention for an anterior midline electrode group (the electrodes used are highlighted in red in the side plot). (a) Mean time-lagged cross-correlations between EEG power and visual attention for infant solo play (equivalent to Fig 2A); (b) same plot for infant joint play (equivalent to Fig 3B); (c) same plot for parent solo play (equivalent to Fig 2B); (d) same plot for parent joint play (equivalent to Fig 4B); (e) mean time-lagged cross-correlations between parent EEG power and infant attention for solo play (equivalent to Fig 5A); (f) same plot for joint play (equivalent to Fig 5B). Data underlying this figure can be found in S2 Data. EEG, electroencephalography.
(TIF)

S6 Fig Time-lagged cross-correlations between EEG power and visual attention for a posterior midline electrode group (the electrodes used are highlighted in red in the side plot). Order of plots (a)–(f) is identical to that shown for S5 Fig. Data underlying this figure can be found in S2 Data.
(TIF)

S7 Fig Spectrograms and results of cluster-based permutation tests from split half analyses. Significance values indicate the significance levels of the cluster-based permutation test conducted as described in the main text. Data underlying this figure can be found in S2 Data.
(TIF)

S8 Fig Control analysis conducted to examine the possibility that the lagged cross-correlation observed between infant attention and parental theta activity may be attributable to differences in parents’ own gaze behaviour. (a) is equivalent to Fig 5E in the main text; (b) is equivalent to Fig 6C in the main text. Data underlying this figure can be found in S2 Data.
(TIF)

S1 Table Full results of the linear mixed effects models for Analysis 3. (DOCX)

S1 Text Supplementary Materials and Results. (DOCX)

S1 Data Data underlying the main figures. (XLSX)

S2 Data Data underlying the supporting information figures. (XLSX)

## Abbreviations

EEG: electroencephalography
ICA: independent component analysis
