# Supplementary figures and images for: Parental neural responsivity to infants’ visual attention: How mature brains influence immature brains during social interaction
Source: PLoS Biol. 2018 Dec 13;16(12):e2006328. doi: 10.1371/journal.pbio.2006328 (PMC6292577; doi:10.1371/journal.pbio.2006328)

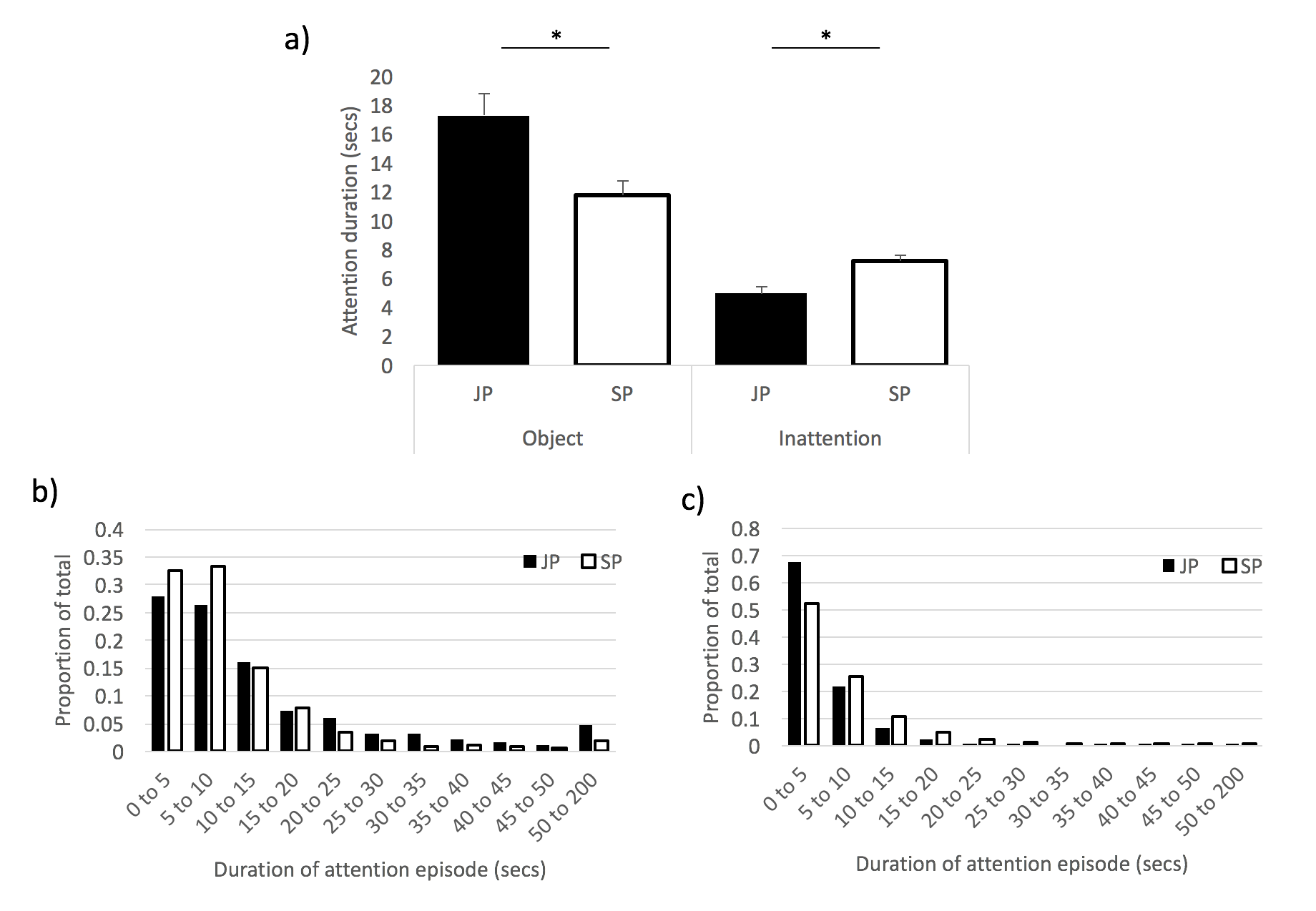

Supplement: S1 Fig — (a) Mean durations of attention episodes towards the object and inattention. Error bars show standard errors. Stars above the plots indicate that attention durations towards the object were found to be significantly longer during joint play than solo play, and episodes of inattention were significantly shorter. (b) Histogram of all attention episodes towards the object in joint play and solo play. (c) histogram of all episodes of inattention in joint play and solo play. Data underlying this figure can be found in S2 Data. (PNG) [file pbio.2006328.s001.png]

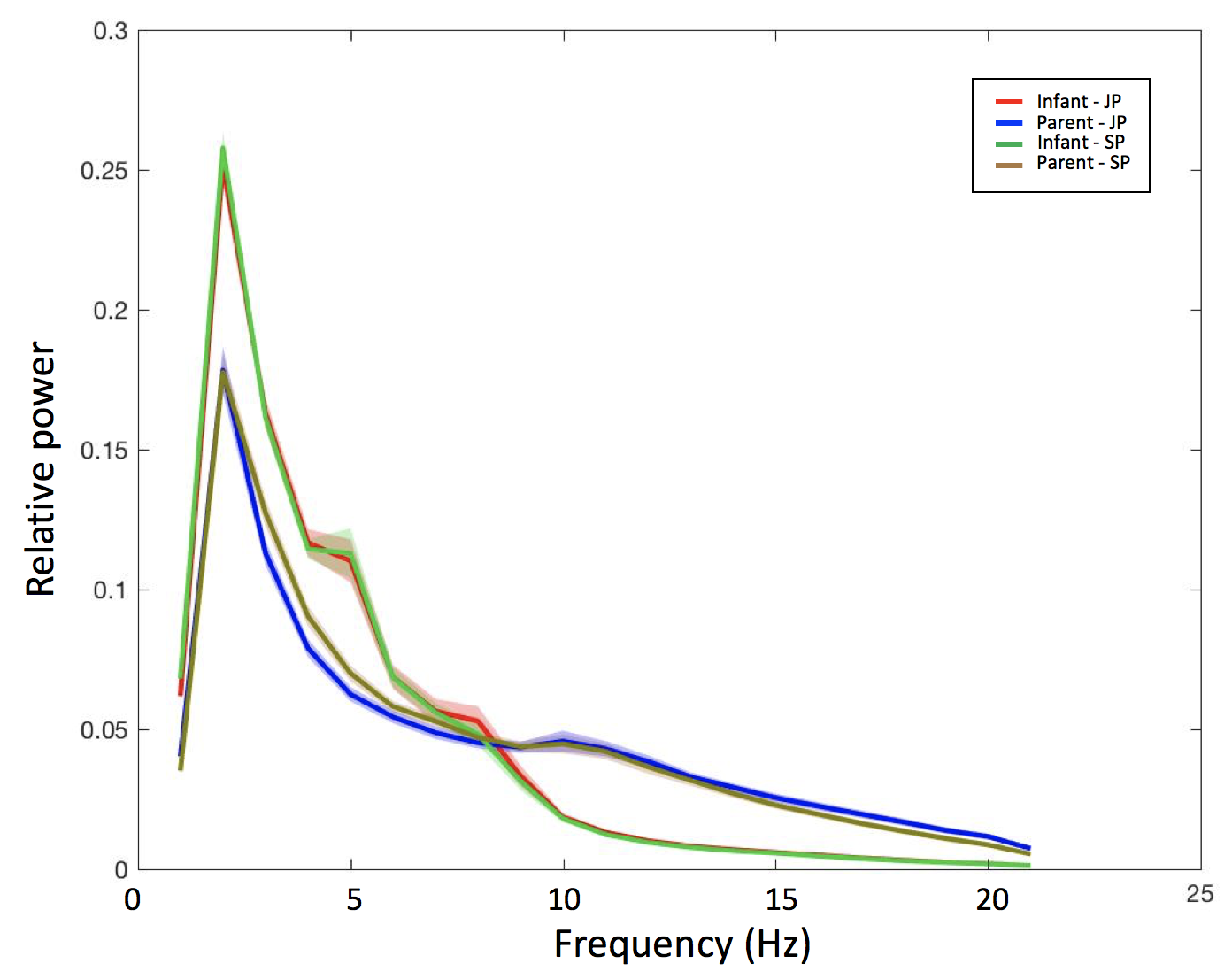

Supplement: S2 Fig — Data underlying this figure can be found in S2 Data. (PNG) [file pbio.2006328.s002.png]

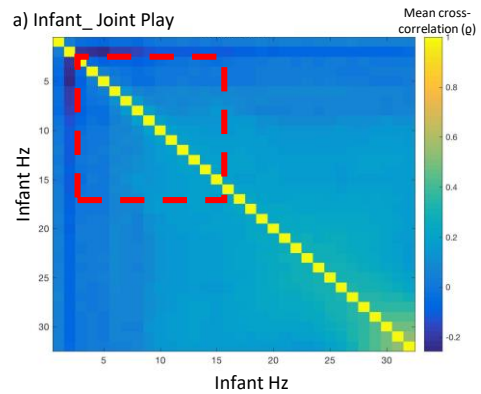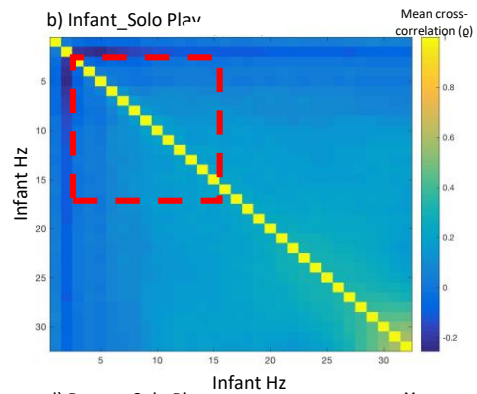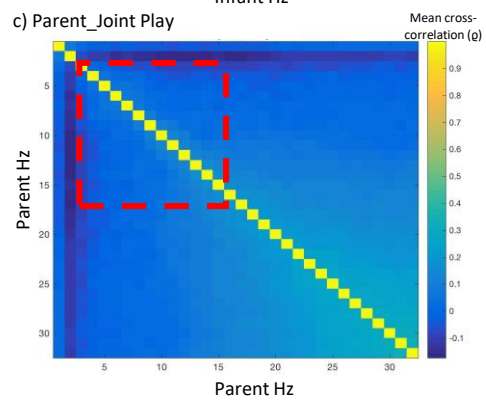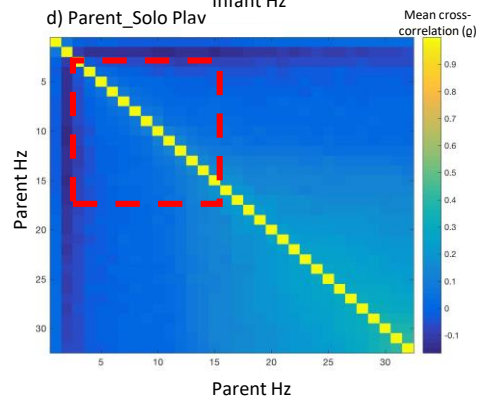

Supplement: S3 Fig — Data underlying this figure can be found in S2 Data. EEG, electroencephalography. (PDF) [file pbio.2006328.s003.pdf]

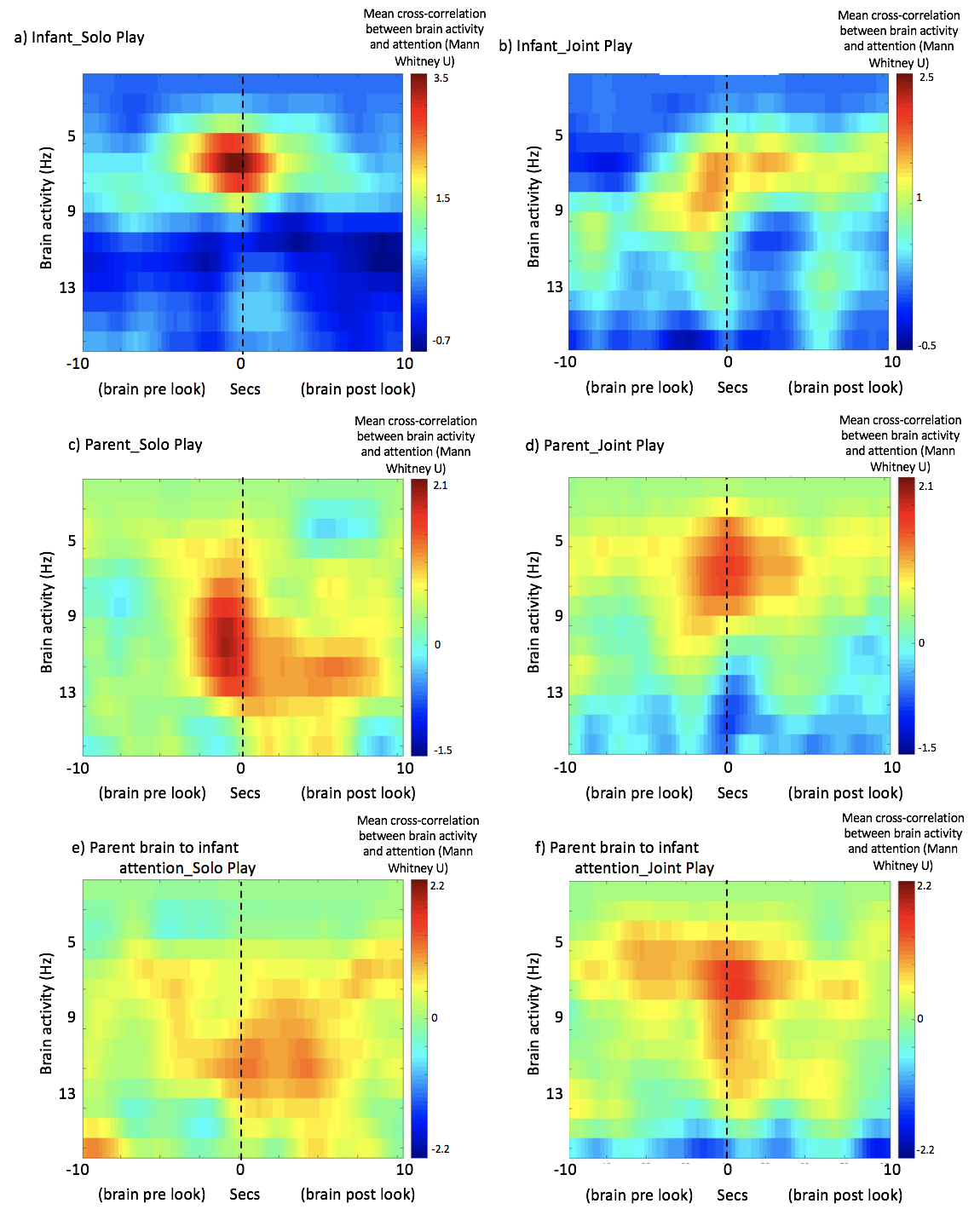

Supplement: S4 Fig — S3A and S3B Fig—equivalent to Fig 3A and 3B. S3C and S3D Fig—equivalent to Fig 4A and 4B. S3E and S3F Fig—equivalent to Fig 5A and 5B. Data underlying this figure can be found in S2 Data. (TIF) [file pbio.2006328.s004.tif]

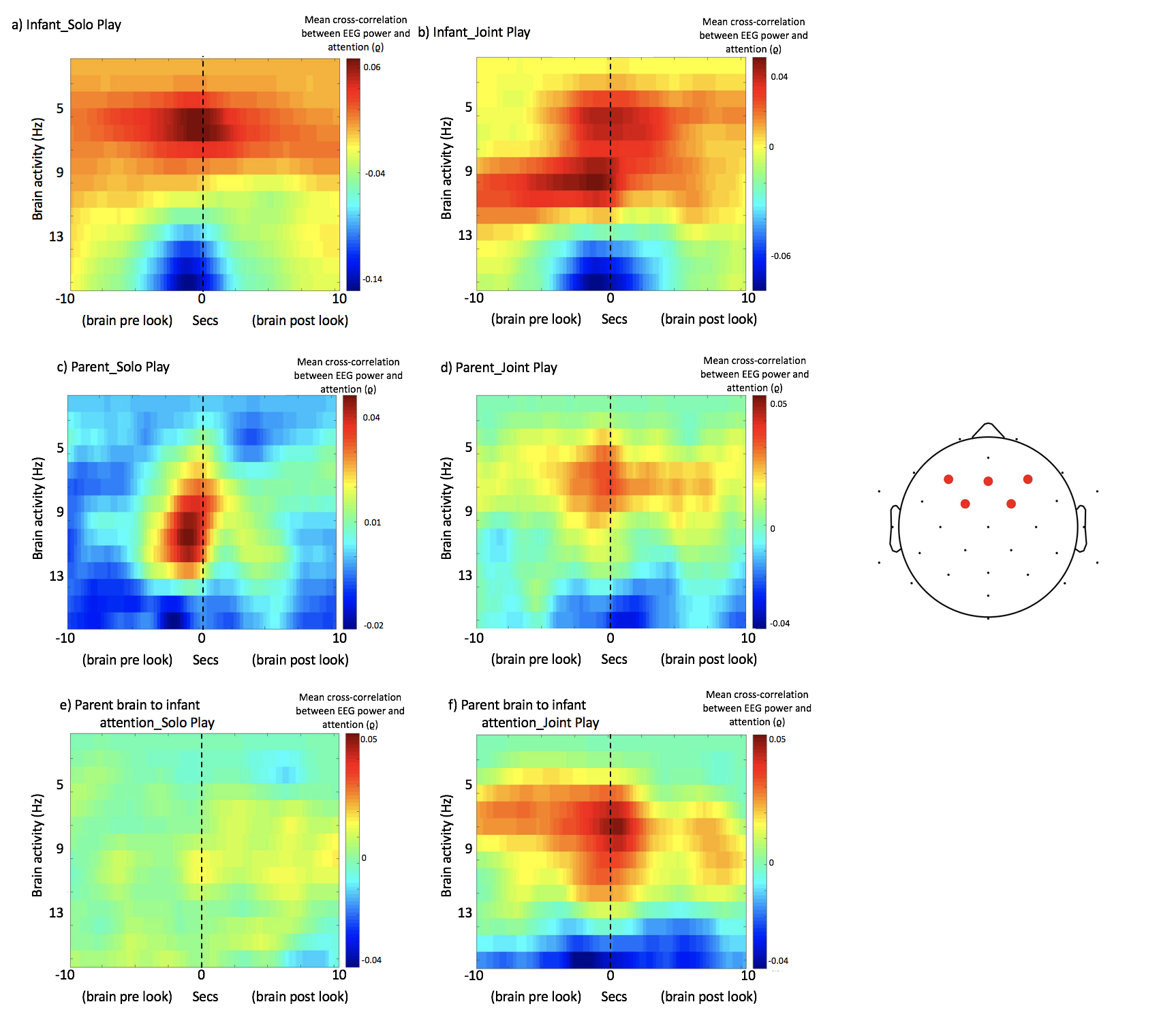

Supplement: S5 Fig — (a) Mean time-lagged cross-correlations between EEG power and visual attention for infant solo play (equivalent to Fig 2A); (b) same plot for infant joint play (equivalent to Fig 3B); (c) same plot for parent solo play (equivalent to Fig 2B); (d) same plot for parent joint play (equivalent to Fig 4B); (e) mean time-lagged cross-correlations between parent EEG power and infant attention for solo play (equivalent to Fig 5A); (f) same plot for joint play (equivalent to Fig 5B). Data underlying this figure can be found in S2 Data. EEG, electroencephalography. (TIF) [file pbio.2006328.s005.tif]

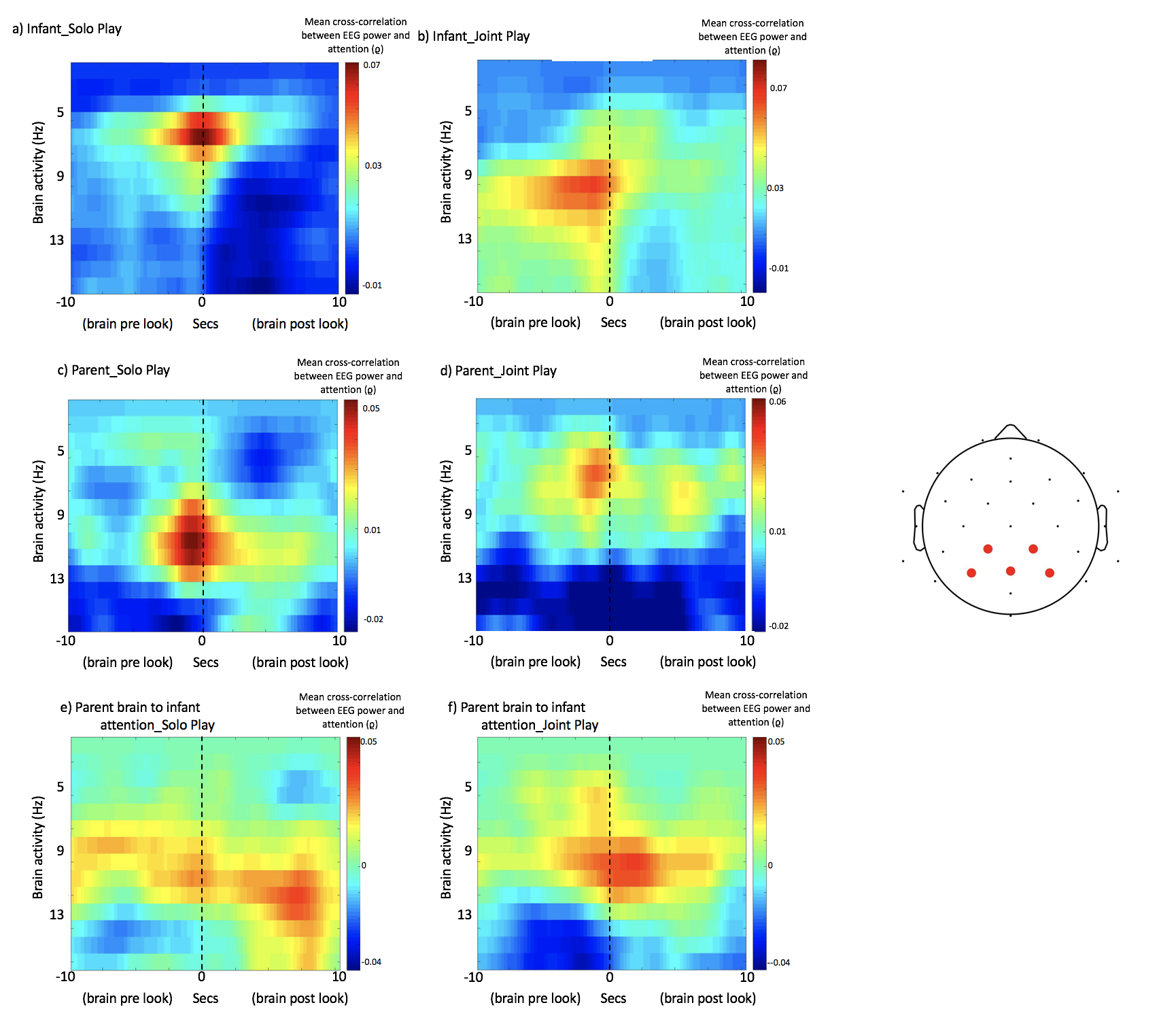

Supplement: S6 Fig — Order of plots (a)–(f) is identical to that shown for S5 Fig. Data underlying this figure can be found in S2 Data. (TIF) [file pbio.2006328.s006.tif]

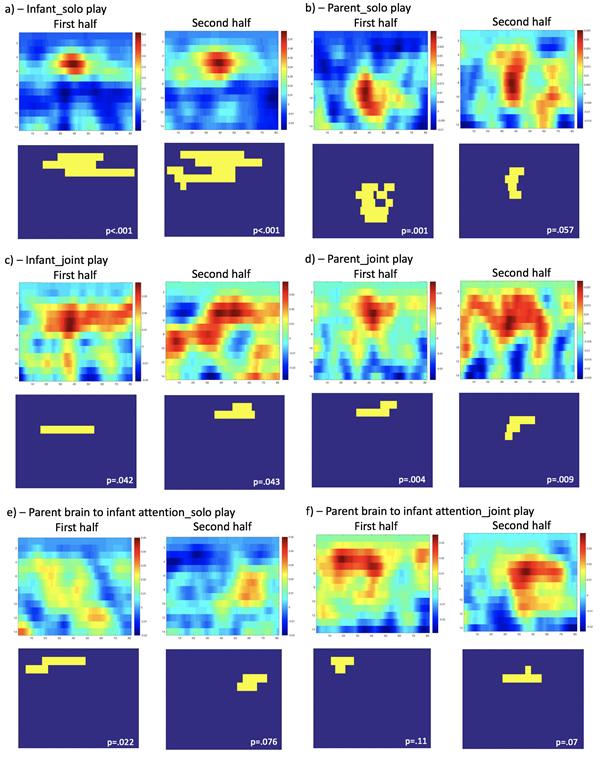

Supplement: S7 Fig — Significance values indicate the significance levels of the cluster-based permutation test conducted as described in the main text. Data underlying this figure can be found in S2 Data. (TIF) [file pbio.2006328.s007.tif]

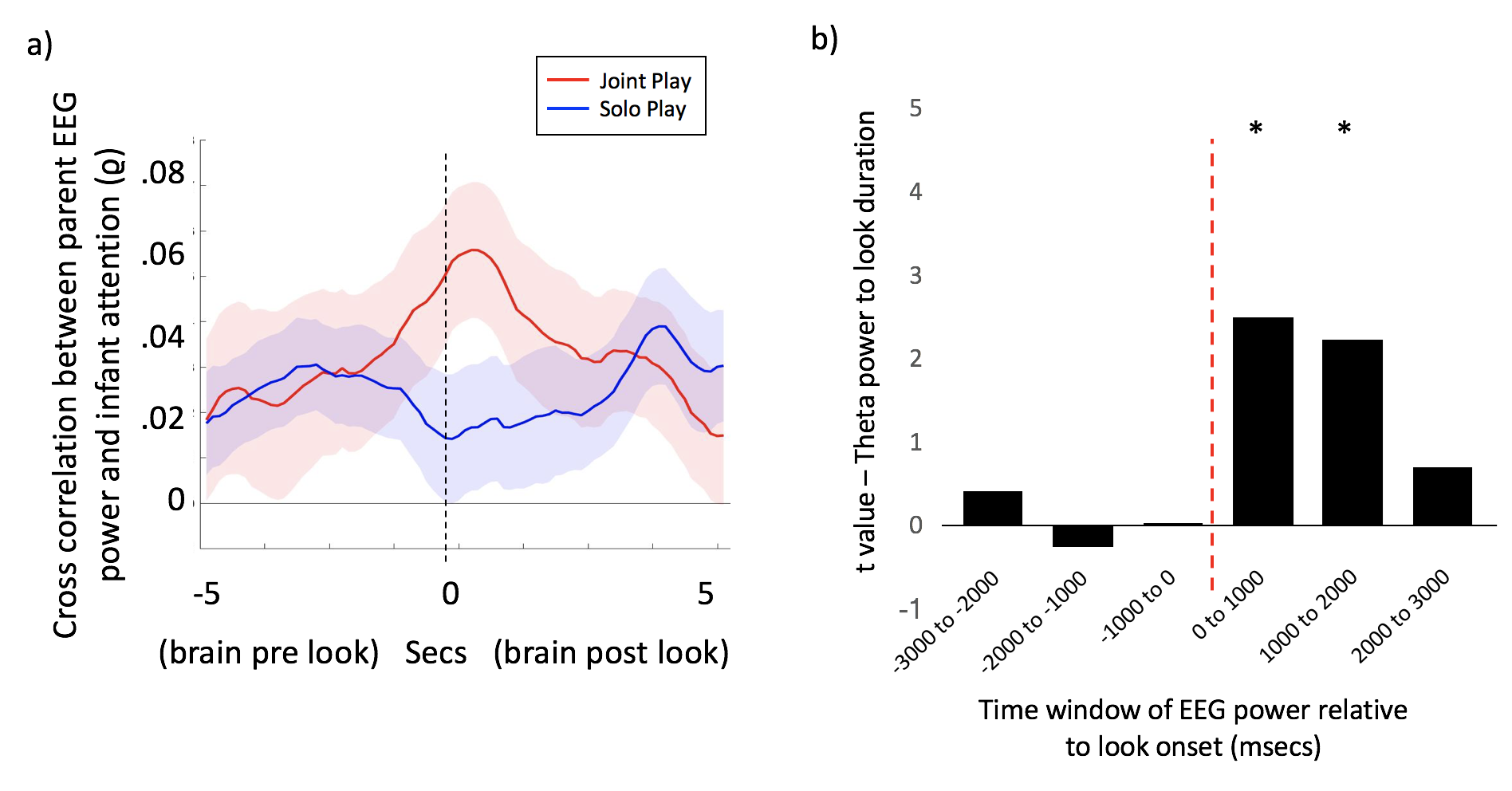

Supplement: S8 Fig — (a) is equivalent to Fig 5E in the main text; (b) is equivalent to Fig 6C in the main text. Data underlying this figure can be found in S2 Data. (TIF) [file pbio.2006328.s008.tif]

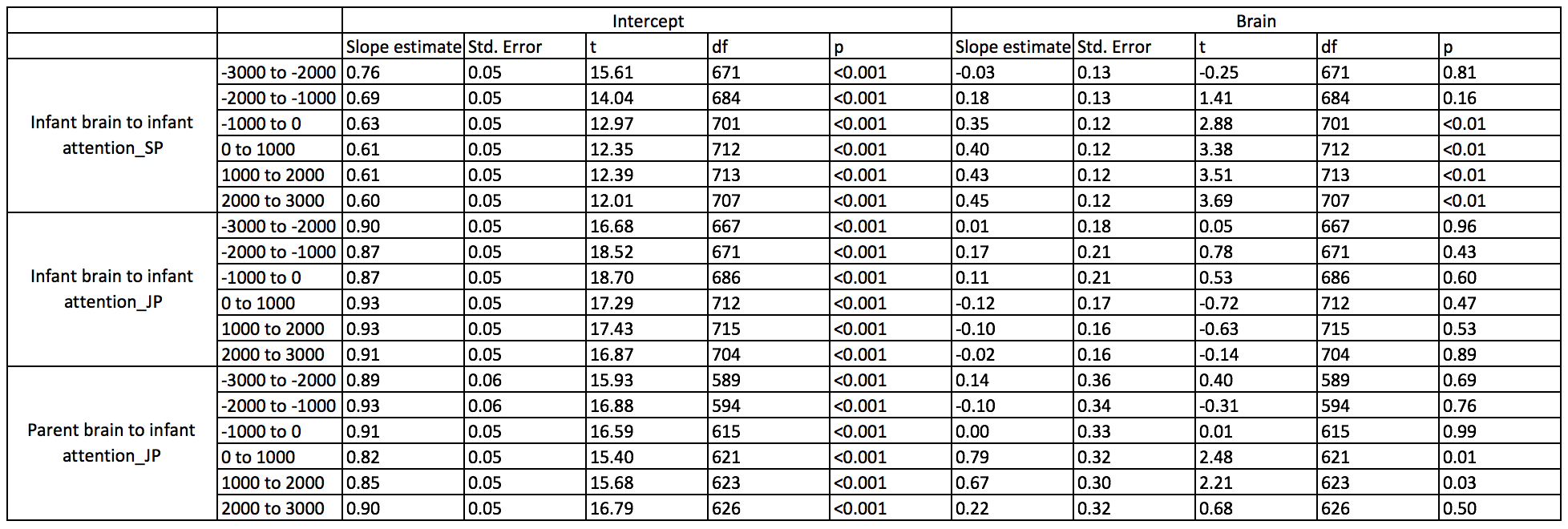

Supplement: S1 Table — (DOCX) [file pbio.2006328.s009.docx]
