# Supplementary material for: Parental neural responsivity to infants’ visual attention: How mature brains influence immature brains during social interaction
Source: PLoS Biol. 2018 Dec 13;16(12):e2006328. doi: 10.1371/journal.pbio.2006328 (PMC6292577; doi:10.1371/journal.pbio.2006328)
Supplement: S1 Text — (DOCX) [file pbio.2006328.s010.docx]

**Supplementary Materials**

**Supplementary Methods**

*1.i Further details on EEG data acquisition*

*1.ii Further details on video coding and synchronisation.*

*1.iii Further details on EEG pre-processing and artefact rejection*

*1.iv Further details on EEG spectral power analysis*

**Supplementary Results**

*2.i Preliminary analyses - look durations*

*2.ii Preliminary analyses – EEG power*

*2.iii Analyses 1 and 2 – cross-spectrum cross-correlations*

*2.iv Analyses 1 and 2 – evaluation of how between-condition differences in artefact rejection rates and toy presentation durations may have influenced primary outcomes.*

*2.v Analyses 1 and 2 – comparative analyses using Mann-Whitney U test*

*2.vi Analyses 1 and 2 – comparative analyses using alternative bootstrapping method*

*2.vii Analyses 1 and 2 – comparative analyses using alternative electrode groupings*

*2.viii Analyses 1 and 2 – comparative analyses using split-half analyses
2.ix Analysis 2 – Control Analysis*

**Supplementary Methods**

*1.i EEG data acquisition.*

EEG signals were acquired using wireless amplifiers to reduce distraction for the infant during testing. EEG was recorded at 500 Hz with no online filtering using AcqKnowledge software (Biopac Systems Inc). Conductive electrode gel SuperVisc (EasyCap, GmbH, Germany) was used to affix the electrodes/cap to the scalp and the electrode impedance was kept below 10 kΩ for infants and 20 kΩ for mothers. A vertex reference location was used because it produces comparable results to other reference sites (Tomarken, Davidson, Wheeler, & Kinney, 1992), and is the least invasive for young infants. The ground electrode was placed on the nape.

*1.ii Video coding and synchronisation.*

Play sessions were videoed using two camcorders positioned next to the child and parent respectively, in order to obtain a frontal head-and-shoulders view of each. Synchronisation of the two camcorders was achieved by placing radio-frequency (RF) receiver LED boxes behind the child’s and parent’s chairs, within view of the camcorders. These RF boxes simultaneously received trigger signals from a single source (a laptop running Matlab) at the start of the testing session, and concurrently emitted light pulses that were visible on parents’ and infants’ camcorders.

In order to assess inter-rater reliability, a 20% proportion of our data were double coded by a second, blinded coder. Cohen’s kappa was calculated to assess inter-rater reliability. This was found to be high for both the SP (mean (*std*)) 0.98 (0.01)) and JP (0.97 *(0.003)*) conditions.

*1.iii EEG pre-processing and artefact rejection*

Prior to artefact rejection data were concatenated across the Solo Play and Joint Play conditions for each participant, in order to ensure that all artefact rejection procedures were applied identically across conditions. First, a band-pass filter was applied to exclude activity below 1Hz and above 16Hz. Second, noisy channels were identified by calculating the power spectrum with Fast Fourier Transform (FFT) and summing the total power across the frequency spectrum. Channels for which the total power was greater than two inter-quartile ranges above the mean total power for all channels were excluded. The mean (st.err.) number of channels excluded in this way was 1.44 (0.22) for infants and 0.18 (0.10) for parents. In addition, the total power across the entire frequency spectrum was visually inspected for each channel at this stage, and data from a further 3 infants were excluded because the total power for all channels was markedly above the average total across all infants, and because visual inspection of the data confirmed that this was not due to factors such as sporadic noisy segments, or to ground noise that could be removed via ICA. (These 3 infants were already excluded prior to calculating the final participant numbers reported in the main Methods section.) Third, continuous data were segmented into two-second epochs, and the most egregious sections of noisy data were excluded prior to running the ICA. In order to ensure that comparable amounts of data were retained for infant and adult participants, this was done by calculating the max-min change on a per-channel, per-epoch basis, across all channels and epochs, and determining what level of this threshold would mean that 6% of data were excluded, separately for infants and parents. This threshold was set, for this coarse, initial rejection stage, at +/- 181μV for adults, and **+/-**617 μV for infants, reflecting a naturally higher amplitude of EEG oscillations in infants (de Haan, 2008)

Fourth, an extended ICA algorithm was then run on the data using the runica algorithm implemented within EEGLAB in Matlab (Delorme, Sejnowski, & Makeig, 2007). The time-courses and spatial distributions of the ICs were visually inspected and the components accounting for ground noise, eye blinks, eye movements and other muscular and movement artifacts were then manually marked and removed (Jung et al., 2000). Fifth, channels that had been excluded at stage three were interpolated using the spherical interpolation function from EEGLAB (Delorme & Makeig, 2004). The mean (*st. err*) (range) of electrodes interpolated was 3.41 *(0.31)* (0-8) for infants and 2.00 *(0.21)* (0-5) for adults. One (never both) of the vertex channels (C3 and C4) used for the main analyses was interpolated for 6 infants and 2 adults. Sixth, a baseline correction was applied by calculating the average value for each epoch and for each channel, and subtracting every individual value within each epoch from that average. Seventh, a second max-min criterion was applied, identical to that applied at stage three but with more stringent criteria. For each epoch and for each channel, the max-min value was calculated. Epochs showing a difference >+/- 80μV were excluded from the adult data. The percentage of epochs excluded at this stage was calculated for the adult data, and the threshold determined such that an identical proportion of samples from the infant dataset were excluded. For the infant data, this threshold value was +/- 196 μV. Eighth, data from all channels other than C3 and C4 were excluded, because our analyses have shown that these are the channels that can be most confidently be said to be free of muscular and movement artefact on our semi-naturalistic table-top play paradigm (Georgieva, Lester, Yilmaz, Wass, & Leong, 2017). In Supplementary Figures S5 and S6 we also present, for comparison, equivalent plots based on anterior and posterior midline groupings of electrodes to those presented in the main text, for C3 and C4.

The mean (*std*) proportion of epochs excluded at artefact rejection was 0.088 (*0.083*) for Infant JP; 0.064 (*0.075)* for Infant SP; 0.16 (0.16) for Parent JP; 0.033 (0.050) for Parent SP. Paired-sample t-tests suggested that the proportion of epochs excluded at artefact rejection did not differ significantly between JP and SP for infants (t=.94, p=.36); but did differ significantly between JP and SP for parents (t=.4.00, p=.001). In section 2.iv we present the results of an analysis conducted to assess whether this difference may have influenced the results of our main analysis.

*1.iv EEG spectral power analysis*

To calculate EEG spectral power, a linear detrend was first applied, for each channel and for each epoch, and then an FFT was carried out using the built-in function in Matlab (Mathworks Inc). The FFT was performed on data in 2000 ms epochs, which were segmented with an 87.5 % (1750 ms) overlap between two adjacent epochs. The FFT was calculated in 1Hz frequency bins, examining frequencies between 1 and 16 Hz. For each epoch, that power at that bin was expressed as relative power – i.e. the total power at that frequency divided by the total power across all frequencies (1-16 Hz) at that epoch. Afterwards, results from the two channels analysed for each participant were averaged. Thus, power estimates of the EEG signal were obtained with a temporal resolution of 4 Hz and a frequency resolution of 1 Hz.

**Supplementary Results**

2.i Preliminary analyses – look durations

A previous report based on these data (Wass et al., 2018), that contained behavioural findings only, reported that infants showed longer look durations towards the object during Joint Play (JP) relative to Solo Play (SP), together with shorter periods of inattention. S1 Fig shows a summary of these findings.

2.ii Preliminary analyses – EEG power

S2 Fig shows a comparison of differences in EEG relative power, for infants and parents, in the Solo Play and Joint Play conditions. First, when comparing the infants and parents, it can be seen that infants appear to show greater relative power at lower frequencies (<8Hz) and less at higher frequencies (>8Hz). This is consistent with previous research (de Haan, 2008). Infants also show marked peaks in theta activity (c. 5Hz) and low alpha (c. 8Hz), whereas adults show a peak in higher alpha (c. 10Hz) (Orekhova, Stroganova, Posikera, & Elam, 2006). When comparing the two conditions, JP and SP, the mean relative powers obtained appear similar across conditions. In order to assess whether any significant differences were present between the two conditions for either infants or parents, separate series of t-tests were conducted for each frequency separately (in 1Hz bins), to assess, for example, whether the average relative power obtained from each individual infant in the 1Hz bin during the JP condition differed significantly from the average relative power obtained from each individual infant in the 1Hz bin during the SP condition. P values obtained were corrected for multiple comparisons using the Benjamini-Hochberg false discovery rate procedure (Benjamini & Hochberg, 1995). No significant differences were observed, for either infants or parents, between the JP and SP conditions (all ps>0.21).

2.iii Analyses 1 and 2 – cross-spectrum cross-correlations

In order to understand the degree to which relationships observed between attention and EEG power at a particular frequency are independent of relationships observed at other frequencies, it is first necessary to examine the degree to which the different frequencies were independent of one another (see S3 Fig). To do this, we repeated the cross-correlation analysis, based on the same data as used in the main analysis. But, instead of analysing the relationship between attention and EEG power at each frequency independently, we instead examined the relationship between the power profile of different individual EEG frequencies. Only zero-lagged correlations were considered.

Results show the frequency range 0-32Hz. The frequency range included in our main results, 2-14 Hz, is highlighted in red. The results show that associations are present at higher frequency bands, suggesting that fluctuations over time across different frequency bands are not fully independent of one another. At low (<2Hz), consistent negative correlations are also observed. Within the frequency range of interest, however, the low cross-correlations observed suggest that fluctuations over time across different frequency bands are independent.

2.iv Analyses 1 and 2 – evaluation of how between-condition differences in artefact rejection rates and toy presentation durations may have influenced primary outcomes.

In section 1.4 of the SM we reported that the proportion of data excluded at artefact rejection did not differ significantly between the JP and SP conditions for the infant data, but did for the parent data. In order to assess whether this factor may have influenced our primary outcome we calculated, for each participant, the difference in proportion of data lost between the JP and SP conditions. For each participant we also calculated the difference in the peak cross-correlation observed between parental theta power and infant visual attention in the JP and SP conditions. We reasoned that, if the smaller cross-correlation effects observed during JP relative to SP were attributable to an increased proportion of data loss during JP relative to SP, then a systematic relationship would be observed at the inter-participant level between these two variables. No such relationship was observed for either the Infant (r=-.16, p=.58) or Parent (r=-.16, p=.53) datasets. This suggests that the smaller cross-correlation effects between parental theta and infant attention observed during JP relative to SP were likely independent of the proportion of data loss during artefact rejection.

In the Methods section in the main text we also report that the mean duration for which each object was presented was lower for the Joint Play than the Solo Play condition. In order to assess whether this factor may have influenced our primary outcome we calculated, for each participant, the difference in mean toy presentation duration between the JP and SP conditions. For each participant we also calculated the difference in the peak cross-correlation observed between parental theta power and infant visual attention in the JP and SP conditions. We reasoned that, if the smaller cross-correlation effects observed during JP relative to SP were attributable to a longer toy presentation duration during JP relative to SP, then a systematic relationship would be observed at the inter-participant level between these two variables. No such relationship was observed for either the Infant (r=.48, p=.11) or Parent (r=.33, p=.24) datasets. This suggests that the smaller cross-correlation effects between parental theta and infant attention observed during JP relative to SP were likely independent of any difference in toy presentation duration between the two conditions.

2.v Analyses 1 and 2 – comparative analyses using Mann-Whitney U test

All analyses were repeated using the Mann-Whitney U test instead of the Spearman’s test (S4 Fig). The results are highly similar. Although not given here for reasons of space the cluster-based permutation test was also repeated for all datasets and the significant pattern of results were identical to the analyses reported in the main text. This suggests that the results obtained in the main text were not specific to the test used to calculate the cross-correlation statistics.

2.vi Analyses 1 and 2 – comparative analyses using alternative bootstrapping method

In order to confirm the results of the significance calculations described in the main text, an additional, bootstrapping analysis was performed. To calculate the cross-correlation values predicted by chance, each time series was randomly shuffled relative to the other time series and the Spearman’s non-parametric correlation was calculated to estimate the bivariate relationship between the two time-series. This calculation was repeated 1000 times for each participant. The 95^th^ centile value of the bootstrap calculations was estimated, participant by participant, and a paired-sample t-test was conducted to assess whether the peak cross-correlation observed in the time windows -2 to +2 seconds exceeded that predicted by chance.

Analysis 1: Consistent with the results reported in the main text, these results suggested that, for Infant Solo Play, a significant cross-correlation was observed between Theta (3-6Hz) power and visual attention t(24)=5.96, p<.001. For Adult Solo Play, a similar significant relation was observed between Low Alpha (6-9Hz) power and visual attention t(24)=2.50, p=.0097. Analysis 2: Consistent with the results reported in the main text, these results suggested that, for Joint Play, a relationship was observed between parental Theta power and infant attention t(19)=1.73, p=.049. For Solo Play, however, no relationship was observed t(24)=1.29, p=.11.

2.vii Analyses 1 and 2 – comparison plots with alternate electrode groupings.

In order to evaluate whether the results obtained were specific to the specific electrode locations used, we repeated our primary analysis with two alternate electrode groupings: an anterior midline grouping (S5 Fig) comprising F3, F4, Fz, FC1 and FC2 and a posterior midline grouping (S6 Fig) comprising CP1, CP2, P3, Pz and P4.

Results observed with these alternate electrode groups are generally highly consistent with the results just from C3 and C4 presented in the main text. For the anterior electrode groupings, oculomotor and other movement artifacts are present in the data (Figure S5a and S5b); these are absent in the readings at C3 and C4 presented in the main text, and in the data from the posterior electrode groupings (S6a Fig and S6b Fig). This is to be expected given that separate analyses suggested that these types of artifacts were least pernicious for electrodes at vertex locations, as compared to more anterior electrodes (Georgieva et al., 2017). The equivalent plots from adults show no equivalent levels of corruption (S5c Fig, S5d Fig), suggesting that muscular artefact corruption may be more widespread in infant data. Also of note, the finding reported in Figure 5b in the main text, that parental theta activity tracked and responded to changes in infants’ attention, appears marginally more prominent for the anterior midline grouping (S5f Fig) than the posterior midline groups (S6f Fig), suggesting that the source may be more anterior. Future work should, however, investigate this issue in more detail.

2.viii Analyses 1 and 2 – comparative analyses using split-half analyses

In order further to confirm the results of our main analyses, a split half analysis was conducted (S7 Fig). Results were subdivided by whether they were recorded during the first or second half of each testing session. An identical set of analyses were completed to those described in the main text. Similar patterns of associations were noted when the calculations were repeated independently on the two halves; expected small reductions in p values due to reduced power were observed.

2.ix Analysis 2 – Control Analysis

One possibility we considered to account for the effects demonstrated in Figures 5e and 6c in the main text is that infant attention may (Granger-) cause adult attention, which in turn causes increases in Theta activity in adults. We conducted a control analysis to examine this possibility. The data were coded, look by look. Instances in which the adult was not looking towards the play object at the start of an infant’s look, but joined the infant’s gaze towards the object within 2000msecs of the start of the infant’s look, were excluded. 2000msecs was chosen as the time-frame because this is the time-window within which our main effects were observed (Figure 5e, 6c). The main analyses were then repeated, exactly as described in the main text. S8 Fig shows the results. These were identical to those described in the main text. This suggests that the association identified between infants’ attention and adults’ Theta activity is not attributable to the possibility that infant attention may (Granger-) cause adult attention, which in turn causes increased Theta activity in adults.

**References**

Benjamini, Y., & Hochberg, Y. (1995). Controlling the false discovery rate: a practical and powerful approach to multiple testing. *Journal of the royal statistical society. Series B (Methodological)*, 289-300.

de Haan, M. (2008). *Infant EEG and Event-Related Potentials*: Psychology Press.

Delorme, A., & Makeig, S. (2004). EEGLAB: an open source toolbox for analysis of single-trial EEG dynamics including independent component analysis. *Journal of neuroscience methods, 134*(1), 9-21.

Delorme, A., Sejnowski, T., & Makeig, S. (2007). Enhanced detection of artifacts in EEG data using higher-order statistics and independent component analysis. *NeuroImage, 34*(4), 1443-1449.

Georgieva, S., Lester, S., Yilmaz, M., Wass, S., & Leong, V. (2017). Topographical and spectral signatures of infant and adult movement artifacts in naturalistic EEG. *bioRxiv*, 206029.

Jung, T.-P., Makeig, S., Humphries, C., Lee, T.-W., Mckeown, M. J., Iragui, V., & Sejnowski, T. J. (2000). Removing electroencephalographic artifacts by blind source separation. *Psychophysiology, 37*(2), 163-178.

Orekhova, E. V., Stroganova, T. A., Posikera, I. N., & Elam, M. (2006). EEG theta rhythm in infants and preschool children. *Clinical Neurophysiology, 117*(5), 1047-1062. doi:10.1016/j.clinph.2005.12.027

Tomarken, A. J., Davidson, R. J., Wheeler, R. E., & Kinney, L. (1992). Psychometric Properties of Resting Anterior EEG Asymmetry: Temporal Stability and Internal Consistency. *Psychophysiology, 29*(5), 576-592.

Wass SV, Clackson K, Georgieva SD, Brightman L, Nutbrown R, Leong V. Infants’ visual

sustained attention is higher during joint play than solo play: is this due to increased endogenous attention control or exogenous stimulus capture? Developmental Science. 2018. Online early e12667.
